# Cariostatic Effect of Green Tea in Comparison with Common Anticariogenic Agents: An *in Vitro* Study

**DOI:** 10.15171/joddd.2015.009

**Published:** 2015-03-04

**Authors:** Mina Jazaeri, Farzaneh Pakdek, Loghman Rezaei-Soufi, Hamidreza Abdolsamadi, Nasrin Rafieian

**Affiliations:** ^1^Assistant Professor, Department of Oral Medicine, Hamadan University of Medical Sciences, Hamadan, Iran; ^2^Assistant Professor, Department of Oral Medicine, Tabriz University of Medical Sciences, Tabriz, Iran; ^3^Associate Professor, Department of Operative Dentistry, Member of Dental Research Center, Hamadan University of Medical Sciences, Hamadan, Iran; ^4^Associate Professor, Department of Oral Medicine, Member of Dental Research Center, Hamadan University of Medical Sciences, Hamadan, Iran

**Keywords:** Cariostatic agent, dental caries, mouthwash, polyphenols, green tea

## Abstract

***Background and aims.*** Anticariogenic effects of different mouthrinses have been shown previously. In this in vitro study the anticariogenic effects of polyphenol extract of green tea with 0.05% fluoride, 0.2% chlorhexidine and fluoride-chlorhexidine were compared.

***Materials and methods.*** This in vitro study was performed on 50 maxillary premolars in 5 groups: 1) normal saline; 2) a 10% solution of green tea polyphenol extract; 3) 0.05% fluoride; 4) 0.2% chlorhexidine; and 5) fluoride-chlorhexidine. Each tooth was placed in a tube which contained a cariogenic solution. Every day the teeth were washed (depending on the experimental groups) with 5 mL of mouthrinse solution. The depth of the caries was measured under a polarized light microscope. Data were analyzed using SPSS 13.0 with Kolmogorov-Smirnov, one-way ANOVA and Tukey tests.

***Results.*** The mean and standard deviation (in µm) of caries depth were 194±16.43, 175±17.94, 142±9.34, 155±13.27, and 144±8.57 in groups 1 to 5, respectively, with significant differences between the groups (P<0.001). Tukey test showed that although there was no significant difference in the depth of caries in groups 1 and 2 (P>0.001), they were significantlyless than those in groups 3 to 5 (P<0.001). There was no significant difference between decay depth of groups 3, 4 and 5 (P>0.001).

***Conclusion.*** The anticariogenic effect of fluoride-chlorhexidine was the highest among the groups. Although green tea showed higher cariostatic effects than normal saline, in comparison with other mouthrinses, it is less effective. More re-search is strongly recommended for clinical use of green tea as an anticariogenic agent.

## Introduction

One of the most common chronic diseases in the world, dental caries, is an infectious disease caused by the colonization of bacteria, which begins with the decalcification of the non-organic part of the tooth and is followed by the decay of the organic matrix. Despite the vast decrease in dental caries prevalence and its intensity, millions of children and adults still experience tooth loss and malocclusion.^1^ Although great efforts have been made in Iran to decrease dental caries, its prevalence is still high.^-4^ As a supplementary tool alongside mechanical methods such as brushing and flossing, mouthrinses have an important role in the reduction of bacterial counts in the mouth, including *Streptococcus mutans* and consequently, in decreasing dental caries.^5^ Recent research studies have shown that 0.1% solution of green tea could prevent the growth of *Streptococcus mutans *completely and it seems that phenolic compound of green tea extract prevents plaque formation.^6^ A previous study showed that epigalloctachin gallate, one of the polyphenolic ingredients of green tea, could inhibit acid production of *Streptococcus mutans.*^7^ In addition, polyphenolic compounds isolated from green tea suppress the activities of oral bacteria such as *Streptococcus mutans* and *Prevottella gingivalis,* which are strongly implicated in the development of dental caries and periodontal deseases.^8,9^ Apart from an anti-bacterial spectrum, a perfect mouthrinse must have low medicinal resistance and make little contribution to the destruction of oral microflora.^10^ Various studies have proven the effect of different mouthrinses, such as fluoride and chlorhexidine, decreasing dental plaque bacterial counts, including *Streptococcus mutans,* and preventing dental caries.^11^ For instance, it has been shown that fluoride mouthrinses, introduced first in the 1950s subjected to a great deal of clinical research, lead to a 35% decrease in caries based on weekly or daily use.^12^

One of the most common beverages all over the world and particularly in Iran is tea which contains polyphenols, caffeine, flavonols, theine and aromatic compounds.^13^ Herbal polyphenols are, in general, compounds able to precipitate proteins.^14^ Flavone and tea catechin are examples of these polyphenols. The antibacterial qualities of green tea polyphenols have also been proven; scientists have shown that the polyphenols in green tea leaves have preventive effects upon the growth of *Escherichia coli, Streptococcus pyogenes *and *Staphylococcus aureus.*^15^ In 2004, researchers found that the polyphenols in tea are able to control *Streptococcus mutans.*^14^

Given the effect of green tea polyphenols in preventing *Streptococcus mutans,* and the proven effect of *Streptococcus mutans* in bacterial plaques on caries, the use of this common beverage as a mouthrinse can be worthy of research. Since, as our search showed, no research study has been carried out so far on comparisons between the anticariogenic effects of green tea with other mouthrinses on the market in Iran, this study was deemed necessary. This study aimed to compare the anticariogenic effect of green tea polyphenol extract with those of 0.05% fluoride, 0.2% chlorhexidine and the fluoride-chlorhexidine compound in vitro.

## Materials and Methods

In this in vitro study, 50 recently extracted (3 months at most) maxillary premolars with no caries, cracks or previous restorations and perfect enamels (evaluated visually or using magnifying glasses with optical powers of 4) were selected and randomly divided into 5 study groups (N=10).

Group 1 (control): washed with normal saline (Darou Pakhsh Co. Tehran, Iran)

Group 2: washed with a 10% solution of green tea polyphenol (Gulf Supplements, Co., Tehran, Iran)

Group 3: washed with 0.05% fluoride (Namjou Pharmacology Co., Tehran, Iran)

Group 4: washed with a 0.2% solution of chlorhexidine (Namjou Pharmacology Co., Tehran, Iran)

Group 5: washed with a 1:1 solution of 0.2% chlorhexidine and 0.05% fluoride combined^16,17^

Immediately after extraction, the teeth were placed in 0.9% normal saline solution (Darou Pakhsh Co., Tehran, Iran) and then kept in 10% solution of formalin (Darou Pakhsh Co. Tehran, Iran) for disinfection.^18^ After a week, the remaining soft dental tissue was removed using periodontal curettes (Juya, Kashmir, Pakistan) and cleaned by means of fluoride-free pumice (Golchadent Co. Karaj, Iran) and rubber caps (manufactured by Kerr, California, USA). Having covered tooth surfaces – except for a 3×3 mm on the buccal surface – with nail polish, the teeth were placed in an autoclave (Farazmehr, Esfahan, Iran) for sterilization at 121°C and a pressure of 15 pounds for 15 minutes. Then, the tooth apexes were sealed using sticky wax (Azarteb Co, Tabriz, Iran) and then sterilized for 2 hours at a 30-cm distance using UV radiation (Jaltajhiz Co., Karaj, Iran). A brain-heart infusion broth (BHI Broth) (Merck Co., Frankfurt, Germany) was then provided and sterilized by means of an autoclave. The prepared teeth were placed separately in a tube containing 5 mL of brain-heart infusion broth and then placed inside an incubator (Thel Co., Chicago, USA) at 37°C for 24 hours. Afterward, the tubes were visually inspected for turbidity to make sure no contamination existed in the tubes. In order to make the artificial cariogenic media similar to that used in a previous study,^19^ 1.5×10^8^*Streptococcus mutans* cells (equivalent to 0.5 McFarland), 1.5×10^8^ *Lactobacillus* cells (equivalent to 0.5 McFarland) and 3 mL of 20% sucrose solution were added to each tube. For 21 days, the teeth were retrieved once every 24 hours and, based upon the study groups they belonged to, were washed with 5 mL of the study solutions using a syringe.

It is to be noted that the 10% green tea solution was prepared by dissolving 10 g of green tea polyphenol extract in 100 mL of dimethyl sulfoxide DMSO (Sigma Aldrich, St Louis, USA).^17^ Every other day, 2 mL of the solution was removed. To prevent contamination with other bacteria, culture procedures were carried out in agar plates, and 2 mL of fresh solution (containing the cultivated environment and the glucose solution) were replaced by it. After a 21-day period, the teeth were retrieved from the solution and prepared for demineralization depth studies as follows. It should be noted that from this phase on, experiments were blind, and the administrator was not aware which solution was being used.

**Figure 1.**
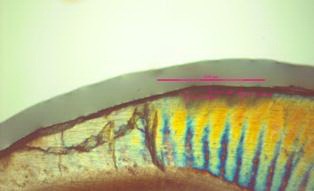
The tooth decay as seen through a polarized light microscope.

After washing in distilled water using the Rotary Microtome Model DS 4055 (Dide Sabz, Uremia, Iran), the teeth were halved buccolingually as cutting passed through the top of the cusps; then two 300-mm sections were prepared from each half. To evaluate the depth of the cavities, the sections were placed under the polarized light microscope BX51-P (Olympus, Tokyo, Japan) with a refraction coefficient of 1.33; the maximum depth of demineralization for each tooth was recorded in micrometers (Figure 1). Data were analyzed using SPSS 13. At first, normal distributions for the groups were assessed using the Kolmogorov–Smirnov test; since the hypothesis for normal distribution for the existing data was not nullified by the Kolmogorov–Smirnov test, one-way ANOVA and supplementary Tukey tests were used at 0.05 level of significance in order to compare the average caries incidence in various study groups.

## Results

**Figure 2.**
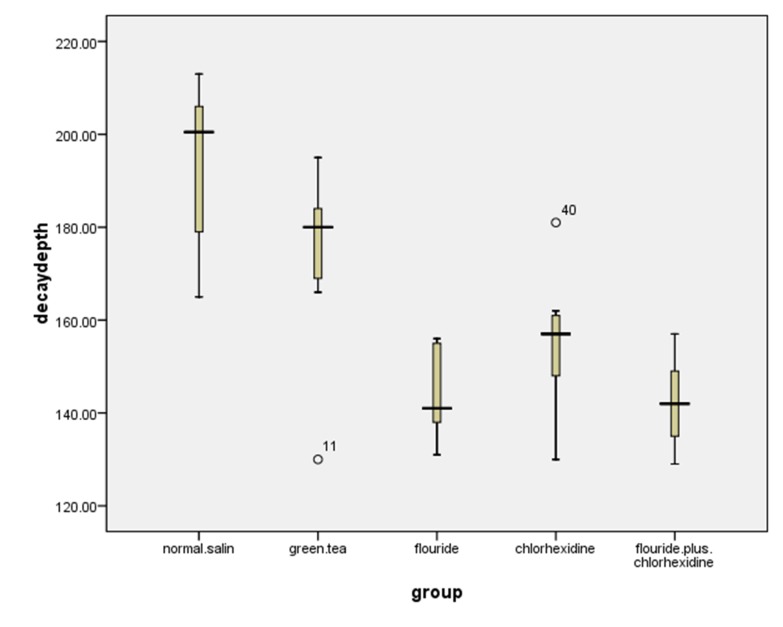
A comparison of decay depth in the presence of the various solutions studied.

The maximum, minimum, mean and standard deviation for the cavity depths of the samples based on study groups are displayed in Table 1. Figure 2 shows the distribution of decay depths for various study groups. Using fluoride and normal saline mouthrinses had the most and least cariostatic effects on enamel, respectively. One-way ANOVA showed a significant difference between the study groups in relation to cavity depths (P<0.001). The results post hoc Tukey tests, presented in Table 2, showed that although the depths of dental caries were not significantly different between groups 1 and 2 (P=0.205), they were significantly deeper in groups 3 to 5 (P<0.001). Tooth decay exhibited no significant differences in groups 3 to 5.

**Table 1:** Decay depth (in micrometers) for various test groups

Washing solution	Mean	Minimum	Maximum	Standard Deviation
Normal saline	194	165.00	213.00	16.43
Green tea	175	130.00	195.00	17.95
Fluoride	142	131.00	156.00	9.34
Chlorhexidine	155	130.00	181.00	13.27
Fluoride- chlorhexidine	144	129.00	157.00	8.57
Total	162	155	169	23.82

**Table 2:** A two-by-two comparison of caries depth in various test groups by means of the Tukey test

Test Groups	Mean differ- ence	P
Normal saline-green tea	19.00	0.205
Normal saline-fluoride	52.00	0.000
Normal saline-chlorhexidine	39.00	0.000
Normal saline-fluoride/chlorhexidine	50.00	0.000
Green tea-fluoride	33.00	0.000
Green tea-chlorhexidine	20.00	0.016
Green tea-fluoride/chlorhexidine	31.00	0.000
Fluoride-chlorhexidine	-13.00	0.384
Fluoride-fluoride/chlorhexidine	-2.00	0.999
Chlorhexidine-fluoride-chlorhexidine	11.00	0.259

## Discussion

Green tea is in fact un-brewed green tea leaves which contain high concentrations of antioxidant and antiinflammatory mediators,^20^ with preventive effects on the growth of* Streptococcus* as shown in 1989 by Toda et al.^21^ Years later, its anticariogenic effect was confirmed in various studies.^22-23^ Polyphenols extracted from green tea are a source of catechin and theaflavin, which prevent the growth and adhesion of *Streptococcus mutans* to tooth surfaces. It seems that this effect is due to the inhibitory effect of polyphenols on the glycosyl transferase of *Streptococcus mutans.*^24^ Since the polyphenol compounds in green tea have been identified as antibacterial compounds,^25^ we attempted in this study to compare the anticariogenic effect of green tea polyphenol extract with that of mouthrinses available on the market (0.05% fluoride, 0.02% chlorhexidine), a combination of fluoride and chlorhexidine and normal saline as the control solution in vitro. The results of this study indicated that 0.05% fluoride solution had the least cavity depth, followed by compound mouthrinses containing fluoride-chlorhexidine, 0.2% chlorhexidine, green tea polyphenol extract and normal saline, respectively, in low depths of cavity. In other words, the depth of the cavities generated after exposing the samples to fluoride-chlorhexidine, 0.05% fluoride and 0.2% chlorhexidine showed a significant statistical difference with the results obtained from green tea polyphenol extract and normal saline; nevertheless, despite the difference in cavity depths, no significant statistical difference was observed between the three study groups that were washed with these three solutions.

In this study, the teeth were exposed to cariogenic environments for 21 days; thus, not only were caries deep enough for histologic study, but also tooth structures were not destroyed so badly that preparing sections would be impossible.^19^ Furthermore, to ensure the fact that the environment was free of any microorganisms that could disrupt the results of the study, a sample of the cariogenic environment was cultivated every two days. In the present study histologic examination which is the most accurate method was used to assess dental caries.

Koyama et al^9^ showed that an individual’s amount of carious lesions correlates with the amount of green tea consumption. Ferrazanno et al^26^ reported that green tea polyphenol prevents dental caries by controlling *Streptococcus mutans* in the oral cavity. Suyama et al^27^ showed that chewing gums containing fluoride extracted from green tea controls acid-caused erosions and dental remineralization. Linke et al^28^ proved that consuming green tea leads to reduced caries in mice fed with cariogenic diets. Subramaniam et al^20^ carried out a study on the effect of green tea on *Streptococcus mutans* and showed that green tea solution prevents its growth more than 0.2% chlorhexidine. As lactobacillus has an important role in dental caries progression, in the present study it was added to the cariogenic solution.

Based on the results of a search carried out in the present study, there are no articles on the comparison of the anticariogenic effect of green tea polyphenol extract with fluoride or chlorhexidine. In contrast to the results of previous studies mentioned above, washing with green tea polyphenol extract did not lead to significant reduction in cavity depths in this study. The reason for the difference in the results from Koyama's^9^ study may lie in the method used such as the fact that green tea was used in a limited manner (once a day) in this research, whereas it was frequently used in Koyama's. Washing with normal saline in the present study is one of the differences between the present study and the previous one. Although normal saline has no anticarious effects its flushing effect probably decreases microbial plaque.^29^ Suyama^27^ used chewing gums containing green tea in his study. Another factor leading to the effectiveness of these gums may be the impact of fluoride, not only the green tea, upon the prevention of cavities. Moreover, the method used in this research—dealing with cavity depths histologically rather than merely studying the clinical effects of these mouthrinses—can also be a reason for the contradiction with the results of previous studies. In any case, future research on the anticariogenic effect of green tea polyphenol extract in samples of higher volumes, higher concentrations of polyphenol extract and frequent use of it seems necessary in order to achieve more accurate results.

## Conclusion

Based upon the results of the present research, polyphenol tea extract, a natural compound, proved to have weaker effects on controlling the formation of caries in comparison to common synthetic compounds used as mouthrinses such as fluoride and chlorhexidine; thus, further studies are required if green tea is to be used as an effective, natural anticariogenic substance. Further studies on other factors which may be effective in dental caries and in vivo study is strongly recommended.

## Acknowledgement

We would like to thank the Research Deputy of Hamadan Medical School for their assistance in this research.
